# Autonomic Dysfunction and Cardiovascular Risk in Patients with Rheumatoid Arthritis: Can Heart Rate Variability Analysis Contribute to a Better Evaluation of the Cardiovascular Profile of a Patient?

**DOI:** 10.3390/jcm12247736

**Published:** 2023-12-17

**Authors:** Elena Esmeralda Saramet, Cristina Pomȋrleanu, Alexandra Maştaleru, Andra Oancea, Doina-Clementina Cojocaru, Mara Russu, Robert Daniel Negru, Codrina Ancuța

**Affiliations:** 1 “Grigore T. Popa” University of Medicine and Pharmacy, 16 Universitatii Str., 700115 Iași, Romania; elena-esmeralda-v-saramet@d.umfiasi.ro (E.E.S.); chirica.mara@d.umfiasi.ro (M.R.); 2 Department of Medical Specialties II, “Grigore T. Popa” University of Medicine and Pharmacy, 700115 Iasi, Romania; daniela.pomirleanu@umfiasi.ro (C.P.); codrina.ancuta@umfiasi.ro (C.A.); 3 Clinical Rehabilitation Hospital, 700661 Iasi, Romania; alexandra.mastaleru@umfiasi.ro (A.M.); andra.oancea@umfiasi.ro (A.O.); doina.cojocaru@umfiasi.ro (D.-C.C.); 4 Department of Medical Specialties I, “Grigore T. Popa” University of Medicine and Pharmacy, 700115 Iasi, Romania

**Keywords:** rheumatoid arthritis, heart rate variability, autonomic imbalance, arrhythmic risk

## Abstract

(1) Background: Rheumatoid arthritis (RA) is a chronic inflammatory disease of autoimmune etiology. Increased scientific evidence suggests that immune-mediated inflammatory dis-eases are associated with autonomic nervous system (ANS) dysfunction. Studies proved that autonomic imbalance is correlated with RA evolution and may explain augmented cardiovascular pathology and mortality not attributable to classical risk factors. (2) Methods: 75 patients (25 males, 50 females) with RA were submitted to standard ECG recording and 24 h Holter monitoring. Twenty-five healthy patients were used as controls. Both time (SDNN, SDANN, SDANN Index, RRmed, rMSSD, and pNN50) and frequency domain (TP, VLF, HF, LF and LF/HF) heart rate variability (HRV) parameters were obtained. Parameters were compared to controls, and correlations with the QTc-interval and inflammatory status expressed through the C-reactive protein (CRP) were evaluated. (3) Results: In patients with a CRP > 5 mg/L, HRV parameters were lower compared to controls and to patients with a CRP ≤ 5 mg/L. All HRV parameters generated by Holter monitoring are negatively correlated with CRP levels and QTc values. The number of premature ventricular contractions (PVC) recorded is correlated with SDNN, SDANN, and LF/HF values. (4) Conclusions: Our study supports recent data suggesting that in RA there is an autonomic system dysfunction strongly connected with the inflammatory status of the patient. The autonomic dysfunction can contribute to the increased risk of cardiovascular death observed in patients with RA.

## 1. Introduction

There is emerging evidence regarding the complex bidirectional link between the autonomic nervous system and the immune system, both of which are involved in the control of inflammation and infection [1,2]. The new concept of “immune-autonomics” reflects the functional and anatomical connections between the two systems [3]. Recent studies have shown that many immunologically mediated chronic inflammatory diseases (including RA) are associated with dysfunction of the autonomic nervous system (ANS) [4,5], and there is increasing interest in evaluating if correction of this dysfunction can be associated with a clinical benefit for patients. Also, there are studies suggesting that ANS dysfunction can precede the development of clinical disease [6]. The vagal nerve has a pivotal role in this dysfunction, and heart rate variability (HRV) is commonly used to evaluate vagal and vegetative dysfunction [7]. HRV parameters can evaluate the vegetative balance based on the vagal and sympathetic effects on cardiac rhythm.

Rheumatoid arthritis is a chronic inflammatory disease with an autoimmune etiology associated with an important public health burden due to important cardio-vascular, psychological, as well as metabolic complications, all driven by persistent inflammation. 

Although disease-modifying antirheumatic drugs (DMARDs), particularly biological agents over non-biologic drugs, may lessen heart risks, patients living with RA still have a twice-higher death risk versus that of the general population, with the excess of mortality mostly explained by cardiovascular diseases [8,9]. Accumulating circumstantial evidence suggests that the higher risk of sudden cardiac death observed in RA cannot be explained only by the early accelerated atherosclerotic process associated with cardiovascular disease; there must be a direct effect of RA on ventricular electric activity and arrhythmic risk [10]. Cumulative exposure to various factors such as augmented myocardial fibrosis deteriorated transiently the outward current and reduced the slow delayed rectifier potassium current induced by pro-inflammatory circulating cytokines that contribute to cardiac electrical instability and sudden cardiac death risk in RA [11].

Signaling a change in the autonomic balance, increased sympathetic and impaired vagal activity may be translated into an increased risk of arrhythmogenesis due to effects on the duration of the action potential [11,12]. Basically, ANS dysfunction, inflammatory status, and cardiovascular risk in RA are interconnected factors that network and picture a pivotal role in the clinical evolution of the patient. The strong relation between reduced vagal activity and cytokines is confirmed [13]. While several studies have confirmed the association between early autonomic imbalance and the clinical and inflammatory status of RA patients [14], there is still a debate over whether ANS abnormal activity is an effect or a contributor to the inflammatory process in RA [4,6]. Remarkably, heart rate variability parameters are associated with CRP levels [15], and there is a positive correlation between the risk of symptomatic coronary artery disease and levels of systemic inflammation biomarkers, particularly the C-reactive protein (CRP) and erythrocyte sedimentation rate (ESR) in RA [16]. 

Cardiovascular autonomic dysfunction can be quantified by measuring heart rate and heart rate variability, while arrhythmic events can be better evaluated using a 24 h Holter electrocardiographic monitoring compared with standard short electrocardiographic recordings regularly used in clinical practice. The analysis and comparison of the HRV parameters from existing studies is difficult due to the important differences in the duration of electrocardiographic recordings used to extract parameters, and usually QT-interval values are not correlated with HRV analysis. Recent data show that there is a circadian variation of both QT-interval and HRV values, which are correlated [16]. This aspect suggests that values of QT-interval and HRV parameters extracted from 24 h recordings can be an optimal solution for analyzing the activity and balance of the autonomic system and QT-interval duration. Our study aimed to investigate the contribution (potentials and pitfalls) of 24 h Holter recordings for the assessment of the relation between autonomic nervous system dysfunction, disease activity, QT interval, and arrhythmic events in patients with rheumatoid arthritis.

## 2. Materials and Methods

### 2.1. Patients and Controls

We conducted a single-center cross-sectional study in a cohort of 75 consecutive RA patients admitted to the Department of Rheumatology at the Clinical Rehabilitation Hospital in Iasi (Romania) between January 2019 and November 2022. All patients were recently confirmed with RA fulfilling the new ACR/EULAR 2010 rheumatoid arthritis classification criteria. RA patients with comorbidities confirmed to interfere with HRV parameters and ECG analysis were excluded: ischemic heart disease, arterial hypertension, cardiac failure, chronic pulmonary diseases, diabetes, liver or kidney disease, atrial fibrillation, bundle branch block, or patients under treatment with beta-blockers or antiarrhythmic drugs. Bundle branch blocks modify QT-interval values, which was the target of another arm of our study. Assessments have been performed before the initiation of DMARDs when patients were only on analgesics (acetaminophen) or non-steroidal anti-inflammatory drugs (NSAIDs). Compared to other studies, our exclusion criteria were set in order to minimize the effect of the elements that have a confirmed influence on HRV parameters. Twenty-five individuals with age, gender, and cardiovascular risk factors matching controls were also asked to participate in the study, after various pathologies and risk factors which can interfere with HRV assessment were ruled out. The study protocol was approved by The Ethics Committee of the Clinical Rehabilitation Hospital of Iasi (Romania); all patients were fully informed, and written informed consent was obtained before enrollment in the study. 

### 2.2. ECG Variables and 24-h-ECG Holter

The biochemical profile was obtained at hospital admission when patients were also subjected to a short ECG recording (10 s) realized using a BeneHeart12 (Mindray) electrocardiograph. The unit realizes the automatic calculation of the QT and QTc intervals routinely used for the cardiovascular evaluation of patients. All the patients included in the study were subjected to a 24 h Holter ECG recording using DM Software USA software and recorders (Cardioscan 12 Premier software version 12.5.0078a Nevada, USA and 300-3A recorders with 10 electrodes and 12 leads). No nicotine or coffee consumption was allowed during the 24 h monitoring. All the 24 h Holter recordings were revised by the same trained author (R.N.), and all the normal beats were confirmed before automatic analysis was realized by the software. After validating the QRS complexes, the software calculates for the entire recording period the following HRV parameters: total power (TP), very low frequencies (VLF), low frequencies (LF), high frequencies (HF), LF/HF ratio, mean RR (mean of the RR intervals), standard deviation of the NN intervals (SDNN), root mean square of RR interval successive differences (rMSSD), numbers of pairs of adjacent NN intervals separated by more than 50 ms (NN50), and percentage of adjacent NN intervals separated by more than 50 ms (pNN50). Holter software automatically calculates the mean value of the QTc interval obtained for all the sinusal beats recorded during 24 h monitoring. For each patient, the number of ventricular events (ventricular premature beats and ventricular tachycardia episodes) was recorded. HRV reference values for the 24 h recordings have been obtained from a control lot, matched for age and gender: 25 healthy adults (15 women and 10 men, mean age 57.36 ± 12.15 years) who requested outpatient consultation for minor complaints. 

### 2.3. Statistical Analysis

Open STAT and PSPP software were used for statistical analysis; data are presented as median and range, or means ± standard deviation (SD). Shapiro–Wilks or Kolmogorov tests were used to evaluate the normal distribution of the parameters, while the t-Student and one-way ANOVA F parametric tests were used for all the parameters with a normal distribution; Mann–Whitney U and Kruskal–Wallis tests were used for non-normal distributions. Correlations were evaluated using Spearman or Pearson coefficients, as appropriate. Statistical significance was set at a *p* value of 0.05 or less. 

## 3. Results

Seventy-five patients (50 females and 25 males) with a new diagnosis of RA were eligible for our study. The mean age of the patients was 58.37 ± 8.68 years. Fourteen patients and 4 controls were confirmed smokers, but they denied any tobacco use during hospital admission. According to the Sharp radiological score for RA, 3 patients (4%) were in stage I, 32 (42.6%) in stage II, 33 (44.4%) in stage III, and 7 (9.4%) in stage IV. For the detection of rheumatoid factor (RF), Waaler-Rose and latex tests were used, and 57 patients (76%) were seropositive. All the patients have undergone a hematology and biochemistry panel (Table 1).

From standard electrocardiographic recordings of the RA patients, we obtained the QTc interval which was automatically calculated by the installed software. The mean value was 402.89 ± 22.69 ms. No statistical difference was observed between patients with normal (0–5 mg/L) or elevated levels of CRP (>5 mg/L). From the 24 h Holter recordings of the RA patients, we obtained the HRV parameters in both the time and frequency domains for the study and control groups (Table 2). Except for total power, there were no statistically significant differences between RA patients and controls.

In the study group, compared to males, female patients had significantly lower values for the following parameters: Total Power, VLF, HF, and SDNN Index, while the maxQTc was significantly higher in female patients. We compared HRV parameters in the study group according to the Sharp radiological score of the RA and the presence of the rheumatoid factor (RF) (Table 3). Only RRmed showed significant statistical differences between radiological stage groups (*p* = 0.047). When tested, no significant correlations were found between HRV parameters and RF levels.

We further divided RA patients into two subgroups according to their CRP level (>5 mg/L and ≤5 mg/L), and we compared the HRV parameters between these two subgroups with controls (Table 4). The value of 5 mg/L was chosen as a reference as it is the upper limit for normality by the analyzer (ERBA XL1000) used in our hospital for the determination of CRP levels. 

A closer look at the study subgroup with a CRP level > 5 mg/L showed that SDNN, SDANN, SDNN Index, RRmed, and VLF were significantly lower when compared with controls. Except for the LF/HF ratio, all the HRV parameters of the subgroup with CRP > 5 mg/L were significantly lower when compared with patients with CRP ≤ 5 mg/L. No statistically significant differences were obtained when we compared patients with CRP ≤ 5 mg/L with controls. For the entire study group, we evaluated the correlations between HRV parameters, CRP level, ESR, and mean QTc interval extracted from 24 h Holter recordings and their statistical significance (Table 5). From the analyzed time and frequency domain HRV parameters, only RRmed presented a significant correlation with ESR (r = −0.342, *p* = 0.0027). 

Except for rMSSD, pNN50, HF, and the LF/HF ratio, all the HRV parameters are negatively correlated with CRP levels. All the HRV parameters (except the LF/HF ratio) are negatively correlated with the QTc-interval mean value obtained from 24 h Holter recordings. No correlations were observed between HRV parameters and QTc values obtained from short ECG recordings. The ventricular premature beats (VPB) were also recorded in RA patients using 24 h Holter ECG recordings (Table 6). No episodes of ventricular tachycardia were recorded during monitoring.

We have further analyzed the correlations between HRV parameters and the number of premature ventricular beats recorded during 24 h Holter monitoring (Table 7).

## 4. Discussion

There is increasing evidence confirming the role of autonomic system dysfunction in the pathobiology of rheumatoid arthritis with and without cardiovascular involvement [4]. Measuring HRV parameters is widely accepted as a valid method to evaluate the ANS balance and cardiovascular risk in many pathological situations [17,18], including RA [19]. We have analyzed heart rate variability (HRV) parameters, QT interval, and arrhythmic events collected from 24 h electrocardiogram recordings of 75 patients diagnosed with AR. We chose this method to ensure that our analysis includes values that mirror the usual daily circadian fluctuations of these parameters [20].

There is general acceptance that HF, pNN50, and rMSSD reflect parasympathetic activity, while LF is mainly influenced by sympathetic tonus, and SDNN is a marker of global autonomic system activity. Furthermore, the LF/HF ratio can be used as a reliable marker reflecting the balance between the two components of the vegetative system [19,21]. 

We used 24 h Holter recordings to obtain HRV parameters in the time and frequency domains in order to explore and debate the dynamics of the relation between autonomic dysfunction, cardiovascular disease, and inflammation in patients living with RA. When comparing HRV parameters acquired from RA patients with data obtained from controls (Table 2), we reported lower SDNN, SDANN, and SDNN Index values but with no statistical significance, confirming the results of the latest studies [22,23]. Also, the study group showed non-significant higher rMSSD and pNN50 when compared to controls, consistent with findings supported by Evrengul et al. [24] and Milovanovic et al. [25] in their papers. Conversely, Adlan et al. found significantly lower values for rMSSD and pNN50 in RA patients compared to controls [26]. In addition, Anichkov et al. detected significantly lower SDNN, SDANN, and rMSSD in RA patients compared to controls using 24 h recordings in a study on 23 women with RA [27]. 

When we compared SDNN and SDANN from female patients included in our study group with controls, we did not find any significant difference (*p* = 0.158 and 0.192, respectively). Our observation differs from the result of Rensburg et al. [28], obtained in a study involving 45 female RA patients, who found significantly lower values for all HRV parameters in the study group when compared to healthy controls. This result can be explained by the fact that in Rensburg’s study, the mean CRP level of the analyzed patients was higher compared to our study (8.59 ± 3.15 mg/L vs. 5.40 ± 5.80 mg/L), suggesting a higher inflammatory status of the patients included. Overall, when we analyzed the entire study group, regardless of the degree of activity of the disease, we could not confirm a significant reduction in HRV parameter values compared to controls. The variability of the results shown above can be partially explained by the different durations of the electrocardiographic recordings used in studies to extract HRV parameters (varying between 1, 5, 10, 15, 30, 60 min, and 24 h), the different periods of the day chosen for the ECG recordings, the equipment used, and the studied group—some studies include only female patients [23,27,28]. Also, the differences in the amplitude of the inflammatory process in the analyzed patients can complicate the comparison of results. Analyzing the rMSSD values in RA patients compared to controls, a meta-analysis from Provan et al. [29] confirmed the cardiac parasympathetic dysfunction associated with RA (rMSSD values were significantly lower in RA patients compared to controls) but also underlined the heterogeneity of the analyzed studies.

We found that RRmed is the only HRV parameter influenced by the radiological score of the patients. Only patients in stages II and IV have significantly lower values compared with stage I (*p* = 0.009 and *p* 0.0001, respectively). Our results confirm the absence of a significant association between surrogates of cumulative disease activity like the degree of articular destructive lesions and HRV observed by previous studies [24,29,30].

Rheumatoid factor (RF) is used as a marker for RA diagnosis, having a high sensitivity but a lower specificity for the disease. Large studies have identified a clear association between the presence of RF and higher levels of disease activity (based on clinical and laboratory assessment using ESR) [31,32]. We found that only the SDNN Index, TP, VLF, and LF have significant lower values in seropositive patients, but we were not able to find any significant correlation between HRV parameters and RF levels as expected. This could be a consequence of the relatively small number of patients included in our study. Except for RRmed, no time–domain HRV parameter significantly correlates with ESR. This result confirms the observations of a previous study that used a 24 h Holter and found no correlation between SDNN, SDANN, rMSSD, and ESR in female patients with RA [27] and of Evrengul et al., who used 1 h recordings and found no correlation between ESR and parameters from both the time and frequency domains [24].

Following the same approach as Lazzerini [33], we divided the RA study group into two subgroups according to their inflammatory status based on CRP levels, using the cut-off level of 5 mg/L. This value was used as the upper limit for normality in our study, following the standards of the analyzer used for the determination of CRP levels. Except for the LF/HF ratio, all the HRV parameters in patients with active disease (CPR > 5 mg/L) were significantly lower when compared with patients with a CRP ≤ 5 mg/L, including all three HRV parameters associated with parasympathetic activity, suggesting there is reduced vagal activity in patients with active RA. It is also useful to mention that the LF/HF ratio in patients with active disease was higher than in controls and in patients with a CRP ≤ 5 mg/L (4.01 ± 2.34 vs. 3.90 ± 2.03 vs. 3.19 ± 1.49), suggesting an excess of sympathetic activity associated with the inflammatory status of the RA patient, even though this increase was not statistically significant. Our results confirm the conclusions obtained by Lazzerini et al., who used three 5 min intervals of electrocardiographic recordings to extract the HRV parameters and concluded that in patients with active RA there is an increased sympathetic outflow correlated with the inflammatory process [33]. 

In our study group, we found that SDNN, SDANN, SDNN Index, TP, VLF, and LF are negatively correlated with CRP levels. Our results are concordant with a recent meta-analysis [34] that confirmed consistent negative associations between SDNN, HF, and markers of inflammation (CRP and WBC showed the most consistent and robust negative correlations across HRV indexes). We confirm the increasing evidence [34,35] for a strong connection between inflammation, the cholinergic anti-inflammatory pathway, and HRV and the use of HRV parameters to evaluate the activity of this pathway. Recent studies analyzing the strong correlation of the HRV parameters with the inflammatory status of RA patients suggest the standard use of HRV to predict the efficiency of the RA treatment [36], with potential savings for the healthcare system [37]. 

In RA patients, the association between systemic inflammation, ANS dysfunction, and increased arrhythmic risk followed by cardiac arrest and quantified by the QT-interval duration can be explained by the changes in ion conductance (secondary to ANS dysfunction) and the direct effect of inflammatory cytokines on ventricular myocytes action potential [11,12,38,39]. Recent studies confirmed the increased risk of cardiac arrest in patients with AR, but only for women with RA, not for men [40]. Except for the LF/HF ratio, our results show a consistent negative correlation between all the HRV parameters and the QTc-interval mean value obtained from all the sinusal beats recorded during the 24 h Holter recording. Our results support the hypothesis that in RA patients autonomic dysfunction with sympathetic predominance can directly affect the QT-interval duration [33]. Also, we find a significant positive correlation between the QTc-interval value obtained from Holter recordings and the CRP levels (r = 0.331, *p* = 0.0037), confirming that the inflammatory process can be directly responsible for the increased arrhythmic risk observed in RA patients. No similar correlations were observed when, in our analysis, we used the QTc interval obtained from the standard electrocardiographic recordings realized at patient admission. This difference in QTc-interval correlations is in concordance with the hypothesis that QTc values obtained from 24 h Holter monitoring are a better tool for QT-interval value characterization, a conclusion supported by a recent study suggesting that long-term QTc fluctuations, with serial QTc-interval measurements, can be better markers to evaluate cardiovascular risk compared to classic QTc determination using short ECG recordings [41]. 

Although there is recent evidence that respiratory diseases have superseded and replaced cardiovascular mortality as a major contributor to excess mortality observed in RA patients [42], previous studies using HRV have shown that in RA patients there is enhanced sympathetic activity [24,33], which could be responsible for the higher incidence of cardiovascular events such as ventricular arrhythmic events and SCD [12]. We found a significant negative correlation between SDNN, SDANN, and the number of VPB recorded during the Holter monitoring period. This observation indirectly supports the hypothesis that autonomic dysfunction could play a direct role in the increased arrhythmic activity and SCD rate noted in RA patients [43] and represents an argument for the use of HRV parameters as predictors together with QT-interval parameters for the cardiovascular risk in RA patients [41]. 

Our study has limitations generated by the relatively small number of patients included in the study and by the possible biases generated by the structure of the control group used to compare the HRV parameters. The large variability in methodology, the lack of normative data regarding HRV, and the differences between clinical and biological elements used to characterize the inflammatory and disease status of the patients included in studies make comparisons difficult, as already mentioned by other authors who reviewed the literature [44]. The 24 h Holter recordings can represent a solution to eliminate the potential differences in results caused by variations in duration and moment of recordings. If further studies are capable of ameliorating the standardization of HRV parameter analysis, it is very probable that in RA patients, the use of HRV will become a very useful tool to evaluate both the clinical status and treatment response. 

## 5. Conclusions

Patients with RA have an autonomic vegetative system dysfunction identified using HRV parameters and characterized by an excess of sympathetic activity, which is correlated to the inflammatory status of the patient. Vegetative dysfunction identified using HRV parameters and inflammatory status strongly correlate with QTc-interval values observed in RA patients. Electrocardiographic parameters obtained from 24 h recordings can be a better tool for the evaluation of autonomic dysfunction and cardiovascular risk in RA patients compared to short electrocardiographic recordings due to their ability to identify the circadian variations of analyzed parameters. 

## Figures and Tables

**Table 1:** Biochemical and hematological profiles of the patients included in the study.

Parameter (SI Units)	Value (Mean ± SD)
Glucose (mg/dL)	99.31 ± 25.11
Uric Acid(mg/dL)	3.95 ± 1.20
Blood Urea Nitrogen (mg/dL)	38.19 ± 12.58
ALAT(U/L)	21.30 ± 8.26
ASAT(U/L)	21.43 ± 4.79
Gamma Glutamyl-transpeptidase (U/L)	29.35 ± 18.92
Total Cholesterol(mg/dL)	188.78 ± 37.43
Triglycerides (mg/dL)	115.53 ± 47.39
Creatinine(mg/dL)	0.79 ± 0.14
Alkaline Phosphatase (U/L)	168.24 ± 66.56
C-reactive protein (mg/L)	5.40 ± 5.80
ESR	34.47 ± 25.31
Leucocytes (×10^3^/mm^3^)	7.39 ± 2.01
Erythrocytes (×10^6^/mm^3^)	4.35 ± 0.48
Hemoglobin (g/dL)	12.72 ± 1.11
Platelets (×10^3^/mm^3^)	304.21 ± 78.02
Lymphocites (×10^3^/mm^3^)	2.08 ± 0.89
Neutrophils (×10^3^/mm^3^)	4.57 ± 1.65
Monocytes (×10^3^/mm^3^)	0.45 ± 0.17
Eosinophils (×10^3^/mm^3^)	0.19 ± 0.14
Basophils (×10^3^/mm^3^)	0.09 ± 0.04

Abbreviations: ALAT—alanyl aminotransferase; ASAT—aspartate aminotransferase; ESR—erythrocytes sedimentation rate.

**Table 2:** Values of the HRV parameters from the time and frequency domains for the study and control groups (presented as mean ± SD or median and range, as appropriate).

HRV Parameter	24 h Holter Recordings in Patients with RA (75 Patients)	24 h Holter Recordings in Controls (25 Patients)	*p*
SDNN (ms)	124.05 ± 33.88	131.16 ± 44.30	NS ^a^
SDANN (ms)	110.09 ± 33.18	117.60 ± 44.60	NS ^a^
SDNN Index	47 (27–94)	58 (36–84)	NS ^b^
rMSSD (ms)	27 (10–75)	25 (17–67)	NS ^b^
pNN50 (%)	6 (0–31)	5 (0–18)	NS ^b^
meanRR (ms)	829.13 ± 104.13	796.78 ± 95.85	NS ^a^
TP (ms^2^)	2051.50 (760–7865.7)	2024.6 (1509.2–5417.6)	0.02 ^b^
HF (ms^2^)	127.8 (21.7–592.2)	152.4 (46.1–286.3)	NS ^b^
VLF (ms^2^)	1700.20 (563.1–6312.2)	1371.9 (508.6–4382.8)	NS ^b^
LF (ms^2^)	384.40 (94.6–1244.9)	543.6 (152.3–804.6)	NS ^b^
LF/HF	3.48 ± 1.86	3.90 ± 2.03	NS ^a^

^a^ Student-*t* test, ^b^ Mann–Whitney U test, NS, non-significant. Abbreviations: SDNN—standard deviation of the NN intervals. SDANN—standard deviation of the adjacent NN intervals. rMSSD—root mean square of RR interval successive differences. pNN50—percentage of adjacent NN intervals separated by more than 50 ms. meanRR—mean of the RR intervals. TP—total power. HF—high frequencies. VLF—very low frequencies. LF—low frequencies.

**Table 3:** Comparison of the HRV parameters from the time and frequency domains according to the type of RA (presented as mean ± SD or median and range, as appropriate).

HRV Parameter	Seropositive RA (57 Patients)	Seronegative RA (18 Patients)	*p*
SDNN (ms)	123.19 ± 33.86	126.78 ± 34.78	NS ^a^
SDANN (ms)	110.16 ± 31.15	109.89 ± 39.94	NS ^a^
SDNN Index	45 (27–94)	58 (28–77)	0.012 ^b^
rMSSD (ms)	27 (10–53)	36 (12–75)	NS ^b^
pNN50 (%)	6 (0–27)	13 (0–31)	NS ^b^
meanRR (ms)	830.88 ± 113.08	823.58 ± 70.95	NS ^a^
TP (ms^2^)	1985 (760–7865.7)	3593 (803–6118.7)	0.004 ^b^
HF (ms^2^)	127 (21.7–592.2)	242.1 (25.1–511.3)	NS ^b^
VLF (ms^2^)	1441.9 (563.1–6312.2)	2356.1 (636.2–4668.7)	0.01 ^b^
LF (ms^2^)	370.9 (94.6–1108.9)	882.5 (137.4–1244.9)	0.02 ^b^
LF/HF	3.26 ± 1.84	4.17 ± 1.77	NS ^a^

^a^ Student-*t* test, ^b^ Mann–Whitney U test, NS non-significant. Abbreviations: SDNN—standard deviation of the NN intervals. SDANN—standard deviation of the adjacent NN intervals. rMSSD—root mean square of RR interval successive differences. pNN50—percentage of adjacent NN intervals separated by more than 50 ms. meanRR—mean of the RR intervals. TP—total power. HF—high frequencies. VLF—very low frequencies. LF—low frequencies.

**Table 4:** Values of the HRV parameters from the time and frequency domains for the two subgroups of RA patients according to their CRP level (cut-off value of 5 mg/L) compared to the control group (presented as mean ± SD or median and range, as appropriate).

HRV Parameter	24 h Holter Recordings in RA Patients with CRP > 5 mg/L (26 Patients)	24 h Holter Recordings in RA Patients with CRP ≤ 5 mg/L (49 Patients)	24 h Holter Recordings in Controls (25 Patients)	Patients with CRP > 5 mg/L vs. Controls *p*	Patients with CRP > 5 mg/L vs. CRP ≤ 5 mg/L *p*
SDNN (ms)	100.15 ± 23.03	136.73 ± 31.93	131.16 ± 44.30	0.031	<0.0001 ^a^
SDANN (ms)	87.38 ± 28.01	122.14 ± 29.34	117.60 ± 44.60	0.039 ^a^	<0.0001 ^a^
SDNN Index	37 (27–68)	49 (39–94)	58 (36–84)	0.02 ^b^	0.0002 ^b^
rMSSD (ms)	19 (10–75)	28 (18–53)	25 (17–67)	NS ^b^	0.0051 ^b^
pNN50 (%)	2 (0–31)	6 (0–27)	5 (0–18)	NS ^b^	NS ^b^
meanRR (ms)	740.40 ± 79.47	876.21 ± 83.07	796.78 ± 95.85	0.025 ^a^	<0.0001 ^a^
TP (ms^2^)	1428.8 (760–4328)	2383.7 (1667.4–7865.7)	2024.6 (1509.2–5417.6)	NS ^b^	0.033 ^b^
HF (ms^2^)	54.5 (21.7–511.3)	157.7 (43.7–592.2)	152.4 (46.1–286.3)	NS ^b^	0.0044 ^b^
VLF (ms^2^)	859.4 (563.1–2399.3)	1810 (835.4–6312.2)	1371.9 (508.6–4382.8)	0.034 ^b^	0.0009 ^b^
LF (ms^2^)	328.2 (94.6–1244.9)	403.3 (211.3–1167.8)	543.6 (152.3–804.6)	NS ^b^	NS ^b^
LF/HF	4.01 ± 2.34	3.19 ± 1.49	3.90 ± 2.03	NS ^a^	NS ^a^

^a^ ANOVA one-way followed by Bonferroni post hoc test. ^b^ Kruskall Wallis followed by Sidak post hoc test, NS, non-significant. Abbreviations: SDNN—standard deviation of the NN intervals. SDANN—standard deviation of the adjacent NN intervals. rMSSD—root mean square of RR interval successive differences. pNN50—percentage of adjacent NN intervals separated by more than 50 ms. meanRR—mean of the RR intervals. TP—total power. HF—high frequencies. VLF—very low frequencies. LF—low frequencies.

**Table 5:** Correlations between HRV parameters, CRP level, and mean QTc value extracted from 24 h Holter recordings and their statistical significance.

	CRP		QTc	
HRV Parameter	r	*p*	r	*p*
SDNN (ms)	−0.464	<0.0001 ^a^	−0.424	0.0001 ^a^
SDANN (ms)	−0.477	<0.0001 ^a^	−0.368	0.001 ^a^
SDNN Index	−0.265	0.02 ^b^	−0.664	<0.0001 ^b^
rMSSD (ms)	−0.197	NS ^b^	−0.535	<0.0001 ^b^
pNN50 (%)	−0.151	NS ^b^	−0.505	<0.0001 ^b^
meanRR (ms)	−0.568	<0.0001 ^a^	−0.622	<0.0001 ^a^
TP (ms^2^)	−0.334	0.04 ^b^	−0.634	<0.0001 ^b^
HF (ms^2^)	−0.191	NS ^b^	−0.640	<0.0001 ^b^
VLF (ms^2^)	−0.231	0.04 ^b^	−0.629	<0.0001 ^b^
LF (ms^2^)	−0.231	0.04 ^b^	−0.604	<0.0001 ^b^
LF/HF	0.110	NS ^a^	0.2	NS ^a^

^a^ Pearson test. ^b^ Spearman test, NS, non-significant. Abbreviations: SDNN—standard deviation of the NN intervals. SDANN—standard deviation of the adjacent NN intervals. rMSSD—root mean square of RR interval successive differences. pNN50—percentage of adjacent NN intervals separated by more than 50 ms. meanRR—mean of the RR intervals. TP—total power. HF—high frequencies. VLF—very low frequencies. LF—low frequencies.

**Table 6:** Ventricular premature beats (VPB) recorded during 24 h Holter recordings in RA patients.

VPB on ECG Holter Recordings	
VPB n/24 h (median-range)	137 (0–18,666)
Patients with VPB n (%)	69 (92%)
Patients with VPB > 1000/24 h n (%)	6 (8%)

**Table 7:** Correlations between HRV parameters and the number of ventricular premature beats (VPB) recorded during 24 h Holter recordings and their statistical significance (only statistically significant results are presented).

	VPB Recorded	
HRV Parameter	r	*p*
SDNN (ms)	−0.290	0.01 ^a^
SDANN (ms)	−0.304	0.008 ^a^
meanRR (ms)	−0.445	0.0001 ^a^
LF/HF	−0.266	0.02 ^a^

^a^ Pearson test. Abbreviations: SDNN—standard deviation of the NN intervals. SDANN—standard deviation of the adjacent NN intervals. meanRR—mean of the RR intervals. HF—high frequencies. LF—low frequencies.

## Data Availability

The data presented in this study are available on request from the corresponding author.
